# The Quality of Medical Care in Low-Income Countries: From Providers to Markets

**DOI:** 10.1371/journal.pmed.1000432

**Published:** 2011-04-12

**Authors:** Jishnu Das

**Affiliations:** 1 World Bank, Washington, D.C., United States of America; 2 Centre for Policy Research, New Delhi, India

## Abstract

Jishnu Das provides a perspective on a research article by Paul Garner and colleagues that reports a systematic review of 80 studies comparing the quality of private versus public ambulatory health care in low and middle income countries.

**Linked Research Article**

This Perspective discusses the following new study published in *PLoS Medicine*:

Berendes S, Heywood P, Oliver S, Garner P (2011) Quality of Private and Public Ambulatory Health Care in Low and Middle Income Countries: Systematic Review of Comparative Studies. PLoS Med 8(4): e1000433. doi:10.1371/journal.pmed.1000433.

Paul Garner and colleagues conducted a systematic review of 80 studies to compare the quality of private versus public ambulatory health care in low and middle income countries.

It is widely believed that people in low- and middle-income countries (LMICs) are in poor health because they cannot reach medical services on time. Predicated on this belief, much of global health policy focuses on the physical provision of goods (clinics, equipment, and medicine) and getting doctors to “underserved” rural areas. Yet, recent evidence shows high utilization rates, even among the poor [1],[2].

While problems of access are certainly salient for particular disadvantaged populations, quality is likely *the* constraining factor for the majority.

The excellent systematic review in this week's *PLoS Medicine* by Paul Garner and colleagues [3] focuses discussion on this critical issue. Their finding of poor quality in both the public and private sectors along different dimensions (competence is similar in both, but the private sector is more patient centered) brings much needed evidence to an ongoing debate. The review reflects a logical initial focus in the literature on individual providers rather than the interactions between providers; going forward, broadening the discussion on quality to health care *markets* can generate valuable insights for policy.

## The Context: Health Care Markets in LMICs Are Incredibly Complex

Typically, households can access multiple providers, ranging from fully qualified public and private sector providers to those without any formal medical training in the private sector. In Delhi, India's capital, there are 70 doctors, most in the private sector, within a 15-minute walk of every household. In the private sector, about half are fully qualified and 10%–15% have no medical training, with a higher fraction of qualified providers in richer neighborhoods [4].

According to a recent report, across rural India, the average household can access 3.2 private, 0.3 public, and 2.3 public paramedical staff within their village [5]. In rural Madhya Pradesh—one of the poorest states in India—households can access 7.5 private providers, 0.6 public providers, and 3.04 public paramedical staff. Of those identified as doctors, 65% had no formal medical training and, of every 100 visits to health care providers, eight were to the public sector and 70 to untrained private sector providers.

Consequently, there is enormous variation in practice-quality within villages and neighborhoods. This variation in quality has implications for a variety of policy decisions ranging from standardization and regulation to medical training. Three steps can help bring evidence to bear on policy discussions.

### Step 1: Documenting Practice-Quality Variation

Providers in the informal sector provide a significant fraction of care in many countries. Yet the review by Garner and colleagues could locate only two studies on quality in the informal sector from *any* LMIC, both of which were subsequently excluded from the review due to limited data. Speculation that the quality of care must be poor among providers in the informal sector is not backed up by comparative evidence with quality in other sectors. As the review points out, the relevant question is: “Quality in the private sector is poor, but compared to what?” Results from Delhi show that low effort reduces the quality of care in the public sector to the level of untrained providers in the private sector [6]. Data on the relative quality of different types of providers could help explain the large market share of informal sector providers and illuminate the trade-off between access and quality with such providers in the market.

### Step 2: Understanding Provider Behavior

What are the implications of practice-quality variation and competition in the health care market for provider behavior and how do different components of quality—competence and effort—relate to each other? Recent research documents a large gap between medical knowledge and practice: doctors, in countries ranging from Tanzania to India to The Netherlands, do a lot less with real patients than they say they would in similar hypothetical scenarios (vignettes) [7]–[9]. This “know–do” gap responds to incentives: it is higher in the public sector where fixed salaries provide poor incentives to exert effort. But, there is also a large know–do gap in the private sector where doctors have full incentives to provide effort.

Because of the know–do gap, medical training has a small impact on the actual care a patient receives; interventions that can induce higher effort have a very high payoff. But what these interventions may be depends on the underlying explanation of the know–do gap—something we know little about.

If this gap reflects shortages in the health care market so that doctors “ration” care to cater to more patients, lower per-patient effort could be consistent with higher patient welfare. But “rationed” care cannot be the entire explanation. In Tanzania, many doctors see five patients in a day—and then spend 3 minutes on each. Consequently, there is no relationship between patient load and quality in the public sector [10]. When researchers sit with doctors, effort immediately increases, leading to improvements in quality *and* patient satisfaction [11]. Why doesn't competition lead to higher effort and better care in markets with many health care providers?

### Step 3: Moving from Provider to Market Quality

The third step translates provider quality to *market* quality. The core issue here is how practice-quality variation impacts patient outcomes. For instance, the impact of poor quality providers on the market for health care will depend on the extent to which their assessments contradict and confuse accurate diagnoses received from better trained physicians. This in turn depends on the confidence that patients place in different doctors.

When provider quality is known, a market with one excellent and one low quality provider may be better than a market with two average providers, because quality differences will be priced into the cost of services. Patients may visit the more expensive, but excellent, doctor for diagnosis and the poor, but low cost, provider for routine tasks. If provider quality is unknown, the (correct) diagnosis by the excellent doctor may contradict a second (but wrong) diagnosis from the poor quality provider, without any guidance for the patient on which diagnosis is likely correct.

In general, quality in the *health care market* differs from the quality of *individual providers*, and patient knowledge of provider quality mediates this difference. To what extent patients are ignorant of doctor quality is an empirically testable hypothesis; preliminary results from ongoing research in Delhi suggest that household assessments of provider quality match up fairly well with quality assessed through independent medical vignettes. How to aggregate provider to market-level quality is a conceptually and empirically open question.

## What Next?

The paper by Garner and colleagues is a wake-up call for the global health community; the review could identify only 80 studies on quality of care across LMICs. Understanding health care in these contexts requires building on such provider-level data to construct market-level aggregates. Such market-level analysis can help answer policy questions ranging from regulatory issues to the trade-off between access and quality.

## Abbreviations

LMIC: low- and middle-income country

## References

[1] Banerjee A, Deaton A, Duflo E (2004). Health care delivery in rural Rajasthan.. Econ and Pol Weekly.

[2] Makinen M, Waters H, Rauch M, Almagambetova N, Bitran R (2000). Inequalities in health care use and expenditures: empirical data from eight developing countries and countries in transition.. Bull World Health Organ.

[3] Berendes S, Heywood P, Oliver S, Garner P (2011). Quality of private and public ambulatory health care in low and middle income countries: systematic review of comparative studies.. PLoS Med.

[4] Das J, Hammer H (2005). Which doctor? Combining vignettes and item response to measure clinical competence.. J Dev Econ Dec.

[5] Centre for Policy Research, The MAQARI Team (2011). Mapping medical providers in rural India: four key trends.

[6] Das J, Hammer J (2007). Money for nothing: the dire straits of medical practice in Delhi, India.. J Dev Econ.

[7] Leonard KL, Melkiory CM, Vialou A (2007). Getting doctors to do their best: the roles of ability and motivation in health care.. J Hum Resour.

[8] Rethans JJ, Sturmans F, Drop R, Van der Vleuten CPM, Hobus P (1991). Does competence of general practitioners predict their performance? Comparison between examination setting and actual practice.. BMJ.

[9] Das J, Hammer J, Leonard K (2008). The quality of medical advice in low-income countries.. J Econ Perspect.

[10] Maestad O, Ole FN (2010). Overworked? On the relationship between workload and health worker performance.. J Health Econ.

[11] Leonard KL (2008). Is patient satisfaction sensitive to changes in the quality of care? An exploitation of the Hawthorne Effect.. J Health Econ.

